# Effect of knee osteoarthritis on the postoperative outcome of proximal femoral nail anti-rotation in the treatment of intertrochanteric fractures in the elderly: a retrospective analysis

**DOI:** 10.1186/s12891-023-07012-6

**Published:** 2023-11-08

**Authors:** Jiaxing Lv, Xiaolong Li, Wenkui Qiu, Jianjun Ji, Lichao Cao, Lei Li, Yihong Zhang, Zhenyan Su

**Affiliations:** Kaifeng Central Hospital, Orthopedic Ward 2, Kaifeng/ Henan, 475000 P. R. China

**Keywords:** Knee osteoarthritis, Intertrochanteric fractures, Proximal femoral nail anti-rotation, Postoperative efficacy, The elderly

## Abstract

**Background:**

The proximal femoral nail anti-rotation (PFNA) is a commonly used internal fixation system for intertrochanteric fractures (IFs) in older adults. Knee osteoarthritis (KOA) is a degenerative lower extremity disease that occurs most frequently in the elderly. Some patients have already had KOA before the IFs. However, whether KOA impacts the postoperative outcome of IFs has not been reported.

**Objective:**

This study aimed to investigate the effect of KOA on the fracture side on the outcome after PFNA for IFs in the elderly.

**Methods:**

Between January 2016 and November 2021, 297 elderly patients treated with PFNA for IFs were enrolled in this study. They were divided into two groups according to the American Rheumatism Association KOA clinical and radiographic criteria: the control group and the KOA group. Intraoperative bleeding, operative time, length of hospital stay, postoperative time out of bed, fracture healing time, postoperative complications, postoperative Harris hip function score, and Barthel ability to daily living Score were compared between the two groups. Follow-up was routinely scheduled at 1, 3, 6, and 12 months postoperatively.

**Results:**

Based on the exclusion criteria, 254 patients who met the requirements were left to be included in this study, including the control group (n = 133) and the KOA group (n = 121). Patients were followed up for a mean of 17.5 months (12–24 months). There was no significant difference between the two groups in preoperative demographic data, intraoperative blood loss, operation time, and length of stay in the hospital. The control group was statistically significant compared to the KOA group in terms of postoperative time out of bed (17.8 ± 4.0 days vs. 19.1 ± 5.8 days), fracture healing time (13.7 ± 2.2 weeks vs. 14.6 ± 3.7 weeks), and postoperative complications (12.8 vs. 23.1%). The Harris hip function score and Barthel ability to daily living score were higher in the control group than in the KOA group at 1, 3, 6, and 12 months postoperatively (the control group: 63.8 ± 10.9, 71.8 ± 10.3, 81.5 ± 8.7, and 91.6 ± 6.3 vs. The KOA group 61.0 ± 10.4, 68.6 ± 9.1, 79.0 ± 9.2, and 88.5 ± 5.9).

**Conclusions:**

In elderly patients with IFs combined with KOA of the fracture side treated with PFNA internal fixation, KOA increases the incidence of postoperative complications of the fracture, prolongs postoperative time out of bed and fracture healing, and reduces postoperative hip function and ability to daily living. Therefore, treating KOA on the fractured side needs to be considered when treating IFs in the elderly.

## Background

Intertrochanteric fractures(IFs) are among the most common fractures of the lower limb in the elderly [1–3]. It is estimated that more than 6.3 million people will suffer IFs by the middle of the 21st century [4]. IFs in elderly patients are characterized by lower limb pain and dysfunction, and bed rest and braking can lead to complications such as lung infections, lower limb venous thrombosis, and bedsores, seriously affecting the quality of life [5, 6]. For elderly patients who can tolerate surgical treatment, surgical treatment should be actively performed to reduce the time spent in bed so that the patient can regain the pre-injury hip function and improve the ability to do daily living as soon as possible [7, 8]. Proximal femoral nail anti-rotation (PFNA) is the most commonly used internal fixation method for the surgical treatment of IFs in the elderly and is suitable for all IFs [9]. PFNA is characterized by minimally invasive repositioning, firm fracture fixation, minimal trauma, and few complications, which create favorable conditions for post-operative rehabilitation of IFs in the elderly [10].

At the same time, it is essential to note that the incidence of knee osteoarthritis(KOA), a degenerative disease of the lower limbs that severely affects the quality of life of patients, is rising with the increasing aging problem [11, 12]. According to epidemiological surveys, the prevalence of KOA in the elderly population over 60 years of age is 23% and is gradually increasing [13]. Some researchers consider KOA an independent factor in developing IFs in the elderly [14]. However, as surgery is currently the mainstream treatment modality for IFs, it is crucial to pay more attention to whether the symptoms of knee pain, swelling, and limitation of movement due to KOA in elderly patients with IFs combined with KOA on the fracture side have an impact on the postoperative recovery of elderly patients treated with PFNA for IFs.We hypothesized that elderly patients with intertrochanteric femur fractures combined with fracture-side KOA treated with PFNA internal fixation would have an increased incidence of postoperative complications, prolonged fracture healing time, and reduced postoperative hip function and daily living capacity.

To test this hypothesis, We collect clinical and imaging findings from elderly patients with IFs combined with KOA on the fracture side and retrospectively analyze the impact of KOA on the postoperative outcome of elderly patients with IFs treated with PFNA.

## Materials and methods

### Study design, the inclusion and exclusion criteria, participants, sampling

**Fig. 1:**
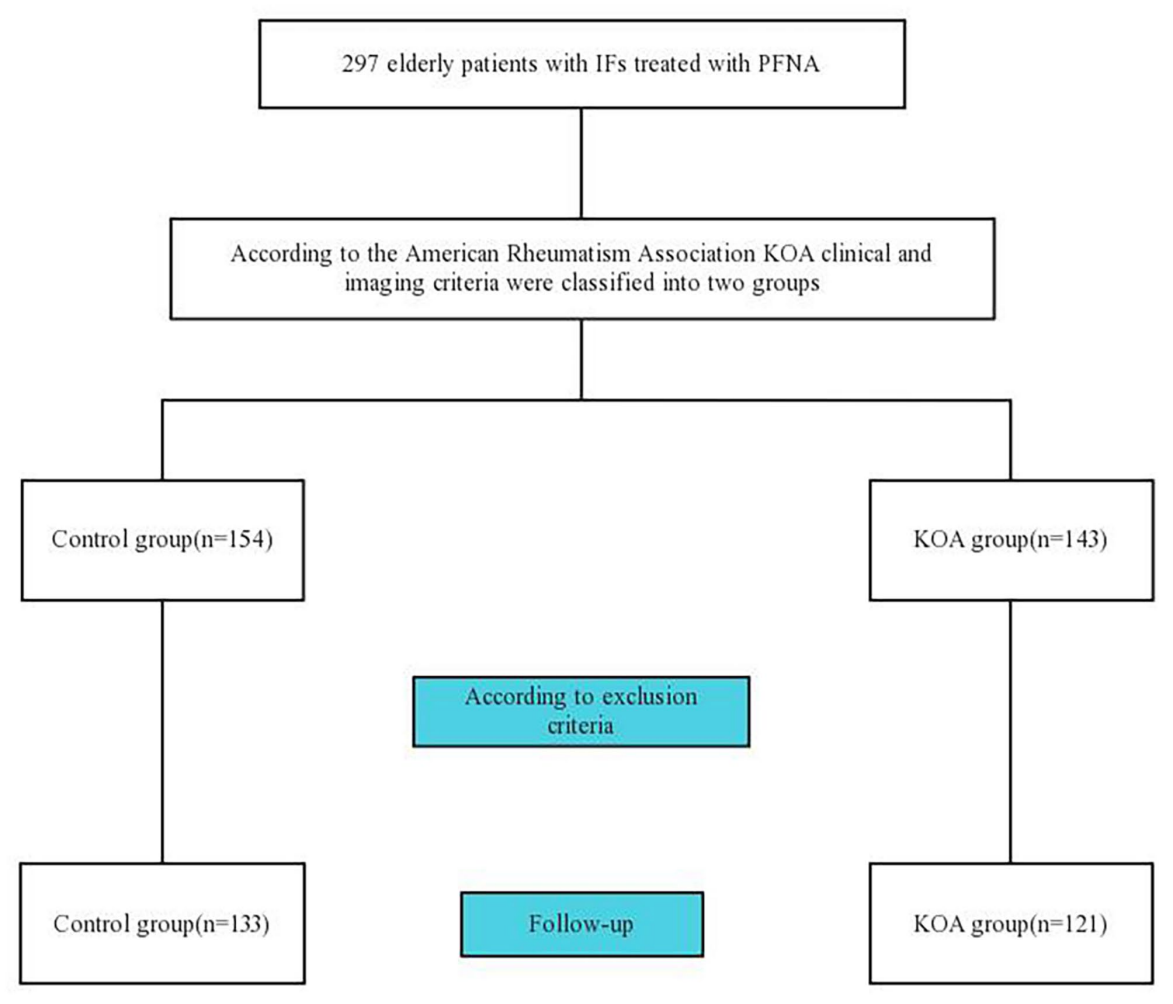
Flow diagram demonstrating methods of studies to investigate the effect of knee osteoarthritis (KOA) on the postoperative efficacy of proximal femoral nail anti-rotation (PFNA) in the treatment of intertrochanteric fractures (IFs) in the elderly

This retrospective study involving human participants were reviewed and approved by the Ethics Committee of Kaifeng Central Hospital (2023ks-lw015) and performed in line with the Declaration of Helsinki international ethical guidelines for studies involving human subjects. The participants provided their written informed consent to participate in this study. Between January 2016 and November 2021, 297 consecutive patients with IFs were treated with PFNA internal fixation. Based on exclusion criteria, 254 patients were included in our study The included subjects were divided into two groups according to the American Rheumatism Association KOA clinical and radiographic criteria [15]: the control group (n = 133) and the KOA group (n = 121). The inclusion criteria were as follows: (1) age greater than or equal to 60 years; (2) unilateral closed IFs; (3) preoperative positive and lateral X-rays of the knee on the fracture side; (4) accepted PFNA internal fixation treatment; (5) no significant skin infection at the surgical site; and (6) complete medical history. The exclusion criteria were as follows: (1) multiple fractures, open fractures, and pathological fractures; (2) combined fractures or injuries at other sites; (3) the previous history of hip fracture; (4) inability to tolerate surgical treatment; (5) other forms of internal fixation; (6) patients with a history of knee trauma, inflammation, and infection and a history of intra-articular intervention and operation; (7) patients with paralysis on the fracture side due to brain disease before the occurrence of the fracture; and (8) death or interruption during follow-up. Demographic characteristics and clinical and radiographic outcomes were recorded. G*Power version 3.1.9.7 was used to calculate the sample size [16]. First, we calculated the effect size of the difference between two independent groups when the sample size was 133:121. Type 1 error probability was set to 0.05 and power to 0.9. In addition, we estimated the sample size for a two-tailed correlation with 0.4 effect size and 0.90 power. A flowchart of the subject inclusion process is shown in Fig. 1.

### Surgical procedure

All patients underwent internal fixation with PFNA performed by experienced orthopedic surgeons specializing in hip surgery with more than 10 years of experience. Cefuroxime 1.5 g was administered intravenously 30 min before surgery to prevent infection. After successful anesthesia (general or spinal), the patients were placed on an orthopedic traction surgical bed. The limb was placed in a neutral position. All patients were closed traction reduction by under C-arm fluoroscopy. The point of entry was slightly medial to the tip of the greater trochanter. A small longitudinal incision is made on the lateral aspect of the greater trochanter and assisted reduction by large-point reduction forceps or Kirschner. The main nail was rotated into the marrow cavity to stabilize the fracture, followed by using a locator to drive the spiral blade proximally and screw in the locking nail distally.

**Fig. 2:**
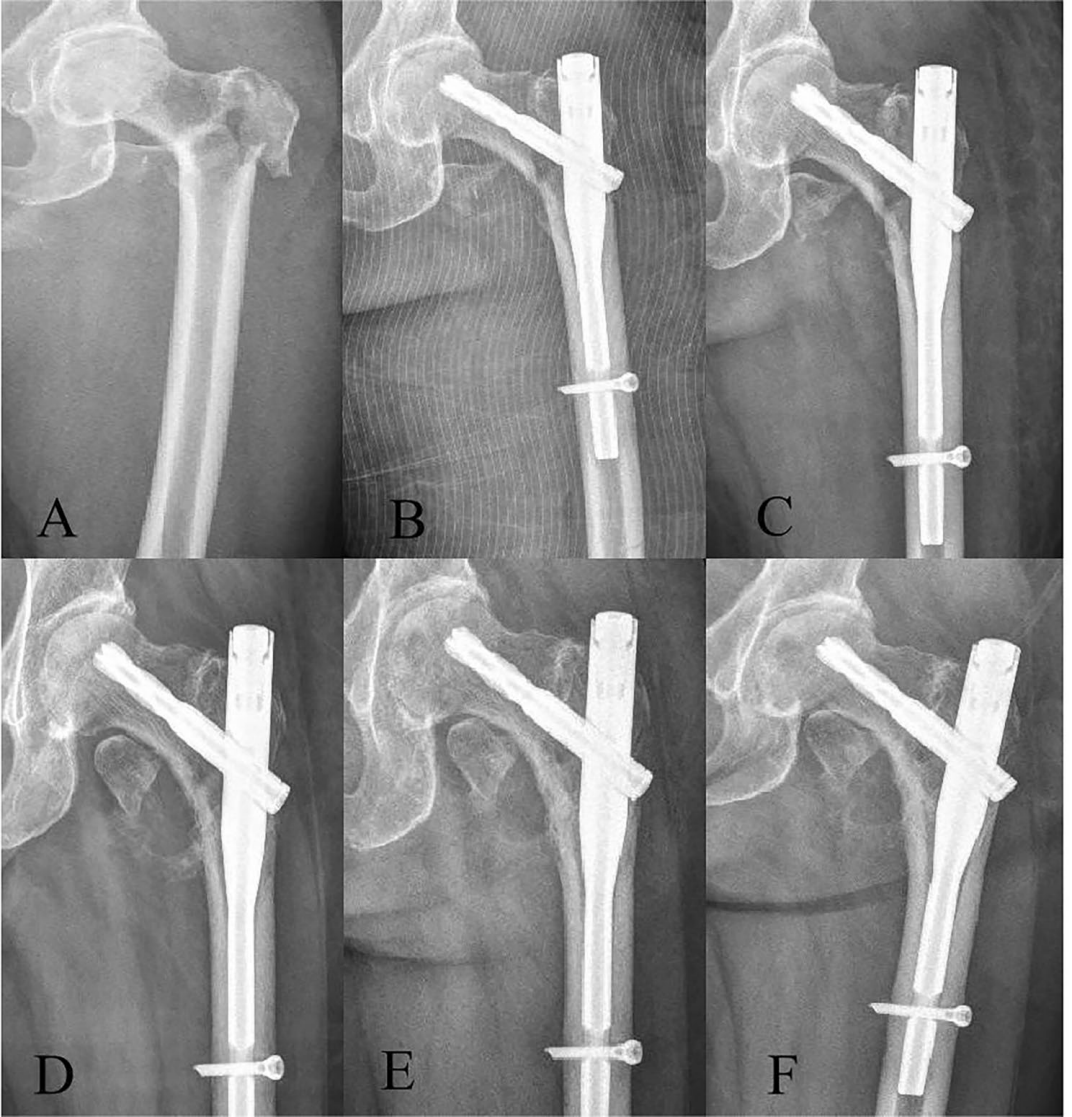
Dynamic observation of X-ray images of fractures. (**A**) Preoperative X-ray; (**B**) postoperative day 1 X-ray;(**C**) 1-month postoperative X-ray;(**D**) 3-month postoperative X-ray;(**E**) 6-month postoperative X-ray;(**F**) 12-month postoperative X-ray

All patients were X-rayed on the first day after surgery to check fracture repositioning and stability. Radiographs were routinely performed at 1, 3, 6, and 12 months postoperatively to evaluate fracture healing (Fig. 2).

### Assessment methods

The Kellgren-Lawrence (K-L) [17] classification system was applied preoperatively to assess the severity of KOA in both groups. The K-L classification system was based on the following five grades of knee X-ray: Grade 0, normal knee; Grade I, suspicious or slight osteophytes without joint space narrowing; Grade II, moderate osteophytes, which may be accompanied by joint space narrowing; Grade III, significant osteophytes with moderate joint space narrowing; Grade IV, large osteophytes with significant joint space narrowing and the presence of subchondral osteophytes.

The postoperative hip function was assessed using the Harris hip function score [18], which includes pain, function, joint mobility, and limb deformity, with a score of 90 or above being considered excellent, 80–89 good, 70–79 moderate, and less than 70 poor. The Harris score provides a more comprehensive assessment of a patient’s functional recovery after hip fracture surgery and is now widely used in clinical follow-up after hip fracture surgery.

The Barthel Index score was used to assess the ability to daily living. The Barthel Index score [19] includes control of urine and stool, dressing, bathing, walking, and so on, with > 60 being good, 60 − 41 being moderate, and ≤ 40 being poor [20, 21]. It assesses the ability to live independently after hip fracture surgery and in KOA patients. Follow-up was routinely scheduled at 1, 3, 6, and 12 months postoperatively. All variables were collected at least 12 months postoperatively.

### Outcome measures

Recorded outcomes included intraoperative bleeding, operative time, length of stay in hospital, postoperative complications, postoperative time out of bed, fracture union time, and Harris score Barthel Index score at 1, 3, 6, and 12 months postoperatively.

### Statistical methods

SPSS software (version 25.0, IBM Crop., USA) was performed for statistical analysis. Descriptive statistics were used to represent clinical case characteristics, including means (SD) for continuous variables and frequencies (percentages) for categorical variables. Independent samples t-tests were used to compare differences in continuous variables between the two groups of patients. The chi-square test was used to compare differences between categorical variables. All comparisons were considered statistically significant at *P* < 0.05.

## Results

### Demographic characteristics and outcomes

The results of comparing the preoperative demographic characteristics of the two groups in Table 1 were not statistically significant (*P* > 0.05). A total of 96 men and 158 women participated in this study. Patients were followed up for a mean of 17.5 months (12–24 months). Patients were divided into 5 categories: 60–69, 70–79, 80–89, and ≥ 90, with a mean age of 75.2 years (60–96 years). The preoperative co-morbidities in both groups were 117 patients. The most common co-morbidities were cardiovascular diseases (16.5%), such as hypertension, and endocrine diseases (11.8%), such as diabetes mellitus. The rest of the patient information is shown in Table 1. Table 2 shows both groups’ K-L grading of the KOA on the fracture side.

**Table 1:** Patient demographics and outcomes in both group

Variable	Control group(n = 133)	KOA group(n = 121)	*P* value
Gender, M/F	50/83	46/75	0.945
Age(years)			0.504
60–69	39	33	
70–79	59	49	
80–89	28	34	
≥ 90	7	5	
Side, L/R	61/72	62/59	0.392
obesity	73/60	72/49	0.458
AO type of fracture			0.376
31 A1	35	24	
31 A2	72	67	
31 A3	26	30	
Comorbidities			0.114
Cardiovascular	20	22	
Pulmonary	13	9	
Digestive	8	9	
Endocrinologic	12	18	
Urologic	2	4	
Time from injury to operation			0.973
< 1 day	25	22	
1–2 days	61	59	
2–3 days	32	27	
> 3 days	15	13	
ASA			0.331
I	31	26	
II	68	52	
III	28	33	
IV	6	10	
Follow up(months)	18.0 ± 3.4	17.5 ± 3.3	0.205

Abbreviation: KOA knee osteoarthritis; ASA American Society of Anesthesiologists

**Table 2:** K-L grade in both group

K-L grade	Control group(n = 133)	KOA group(n = 121)
0	57	0
I	58	0
II	18	11
III	0	65
IV	0	45

Abbreviation: KOA knee osteoarthritis; K-L Kellgren-Lawrence

### Clinical results

There was no statistically significant comparison of intraoperative bleeding, operative time, and length of stay in the hospital between the two groups (*P* > 0.05, Table 3). In the control group, the intraoperative bleeding was (190.1 ± 50.2) ml, the operative time was (63.8 ± 14.0) min, and the hospital stay was (14.0 ± 3.2) days; in the KOA group, the intraoperative bleeding was (196.9 ± 59.7) ml, the operative time was (62.7 ± 19.5) min, and the length of stay in the hospital was (14.6 ± 3.4) days. Postoperative time out of bed and fracture healing were shorter in the control group than in the KOA group (17.8 ± 4.0 days vs. 19.1 ± 5.8 days, *P* = 0.033; 13.7 ± 2.2 weeks vs. 14.6 ± 3.7 weeks, *P* = 0.014). Regarding postoperative complications, patients in the KOA group had a higher rate of postoperative complications than the control group (12.8 vs. 23.1%, *P* = 0.031, Table 4). Among the postoperative complications in the control group, there were 3 cases of pneumonia, 2 cases of venous thrombosis of the lower limbs, 1 case of decubitus ulcer, 3 cases of urinary tract infection, 5 cases of myocardial infarction, 2 cases of secondary fracture, and 1 case of implant loosening; among the postoperative complications in the KOA group, there were 6 cases of pneumonia, 5 cases of venous thrombosis of the lower limbs, 2 cases of the decubitus ulcer, 3 cases of urinary tract infection, 4 cases of myocardial infarction, 2 cases of secondary fracture, 4 cases of implant loosening, and 2 cases of hip inversion.

**Table 3:** Postoperative clinical outcomes in both group

Variable	Control group(n = 133)	KOA group(n = 121)	*P* value
Intraoperative bleeding (ml)	190.1 ± 50.2	196.9 ± 59.7	0.331
Operation time (min)	63.8 ± 14.0	62.7 ± 19.5	0.601
Length of stay in hospital (day)	14.0 ± 3.2	14.6 ± 3.4	0.166
Postoperative time out of bed (day)	17.8 ± 4.0	19.1 ± 5.8	0.033
Fracture union time (week)	13.7 ± 2.2	14.6 ± 3.7	0.014

Abbreviation: KOA knee osteoarthritis

**Table 4:** Postoperative complications in both group

Variable	Control group(n = 133)	KOA group(n = 121)	*P* value
Complication	17(12.8%)	28(23.1%)	0.031
Pneumonia	3	6	
Venous thrombosis of the lower limb	2	5	
Pulmonary embolism	0	0	
Decubitus ulcer	1	2	
Urinary tract infection	3	3	
Myocardial infarction	5	4	
Secondary fracture	2	2	
Loosening of implant	1	4	
Internal hip rotation	0	2	

Abbreviation: KOA knee osteoarthritis

**Fig. 3:**
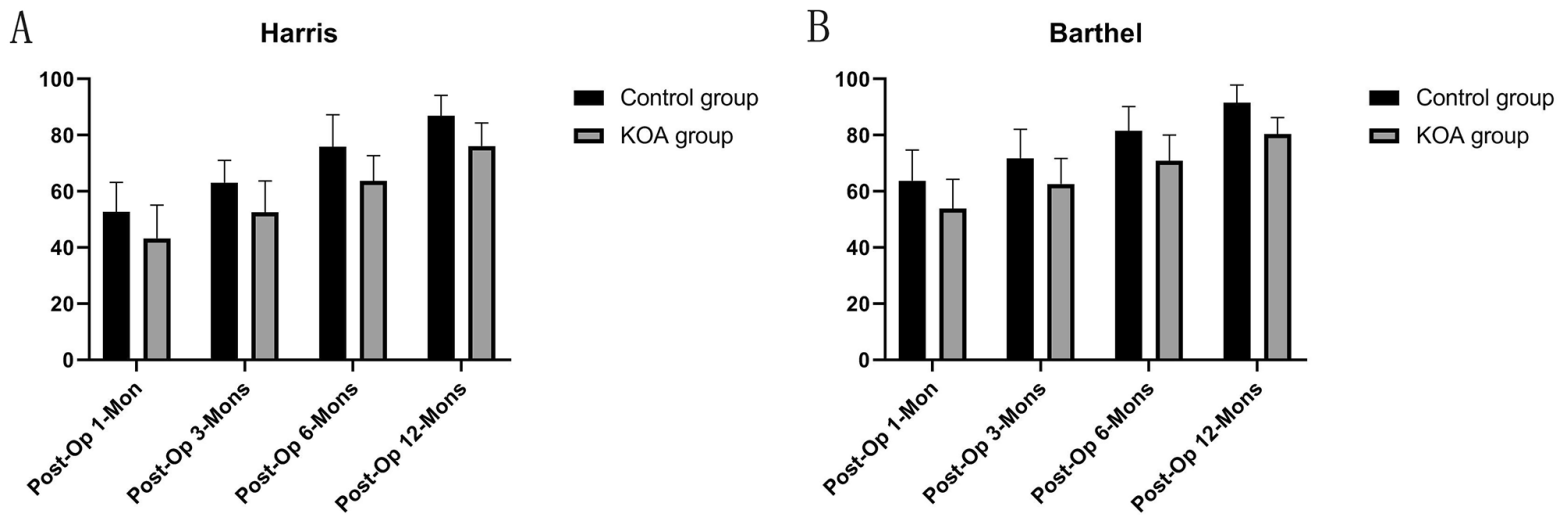
Clinical outcomes at different follow-up time points. (**A**) Harris hip score for hip function. (**B**) Barthel Index score for the ability to make a daily living

The Harris hip function score was used to assess postoperative hip function. A statistically significant comparison of Harris scores at 1, 3, 6, and 12 months postoperatively was made between the two groups (*P* < 0.05, Fig. 3). In the control group, Harris scores were 52.7 ± 10.5, 63.1 ± 7.9, 75.9 ± 11.4, and 86.9 ± 7.3 at 1, 3, 6, and 12 months postoperatively, respectively; in the KOA group, Harris scores were 49.3 ± 11.8, 59.5 ± 11.2, 72.7 ± 9.0 and 84.1 ± 8.3 at 1, 3, 6 and 12 months postoperatively, respectively. The Barthel Index score assesses the patient’s ability to perform postoperative activities of daily living. Barthel Index scores at 1, 3, 6, and 12 months postoperatively were statistically significant in both groups (*P* < 0.05, Fig. 3). In the control group, the Barthel Index scores at 1, 3, 6, and 12 months after surgery were 63.8 ± 10.9, 71.8 ± 10.3, 81.5 ± 8.7, and 91.6 ± 6.3 after surgery, respectively; in the KOA group, the Barthel Index scores at 1, 3, 6 and 12 months were 61.0 ± 10.4, 68.6 ± 9.1, 79.0 ± 9.2, and 88.5 ± 5.9 after surgery, respectively.

## Discussion

IFs, the most common type of hip fracture in clinical practice, can be caused by low-energy injuries and account for approximately 35.7% of hip fractures in the elderly [22]. For IFs in the elderly, the suggestion of orthopedic surgeons is to operate early [23]. Early rehabilitation exercises can be performed after surgery to avoid complications of prolonged bed rest, to improve the ability to perform daily activities, and to return to the patient’s pre-injury level of activity as soon as possible [24, 25].

In the study on the choice of internal fixation for surgical treatment of IFs in the elderly, it was found that PFNA, a modified intramedullary fixation system based on Proximal femoral nail, was currently the preferred internal fixation method for the treatment of IFs [26, 27]. The PFNA has added anti-rotation properties, allowing intraoperative C-arm fluoroscopy for traction closure and repositioning and minimally invasive percutaneous internal fixation with a small incision [28]. Moreover, PFNA was more compatible with the anatomical and biomechanical conduction pattern of the proximal femur and had fewer complications, allowing patients to perform functional rehabilitation at an early stage [29].

KOA is a degenerative disease caused by various factors that lead to fibrosis, cracking, ulceration, and loss of articular cartilage, resulting in joint pain as the main symptom [30–32]. In severe cases, KOA can lead to a lack of muscle strength in the lower limbs, flexion deformities, changes in force lines, and force instability [33].

Prolonged bed rest and reduced activity of the lower limbs increase the incidence of postoperative complications of lower limb fractures [34]. Postoperative lower limb activity is significantly reduced in elderly patients with IFs. The combination of other lower limb diseases on the side of the fracture and prolonged bed rest can dramatically increase the incidence of postoperative complications. KOA is one of the standard lower limb disorders in elderly patients. As the systemic hormone metabolism of elderly patients decreases, the degeneration of the knee joint will gradually increase, resulting in pain, swelling, and limited movement of the knee common, followed by atrophy of the muscles around the joint and joint weakness, resulting in a significant reduction in lower limb motion [35, 36]. In this study, the KOA group had a higher rate of postoperative complications than the control group. It indicated that an elderly IFs combined with ipsilateral knee osteoarthritis does not allow for effective lower limb muscle contraction and joint movement in the affected limb postoperatively. The inability of patients to start upright training early, prolonged bed rest, reduced lower limb activity, and a significant increase in the incidence of postoperative complications seriously affect the postoperative outcome of IFs in the elderly.

In biomechanics, as the human skeleton is mostly irregular in geometry, micro-movement between fracture ends breaks down the axial pressure into various directional components, facilitating fracture healing [37–40]. Micro-movement helps promote osteoblasts’ regeneration at the fracture end and the early formation, development, and maturation of bone scabs [41–43]. Therefore, early out of bed postoperatively for IFs can promote healing of the fracture end and create conditions for early full weight bearing on the floor. In our study, patients in the KOA group had a longer postoperative time out of bed and fracture healing time than the control group. We suggest that KOA, a chronic degenerative condition, and the long-term persistence of pain around the knee joint can cause psychological disorders in patients. It leads to a fear of being out of bed after surgery, a constant decrease in lower limb activity, and the need for psychological support during postoperative rehabilitation. Moreover, the lower limb muscles had atrophied to a certain extent due to the reduced mobility of the lower limb before the injury, resulting in reduced stimulation of the fracture site by the hip muscles, reduced micro-movement of the fracture end, slower growth of the bone scab and longer fracture healing time.

Restoring the function of the affected hip as soon as possible, relieving pain, and improving the patient’s quality of life are the ultimate goals of surgery for IFs in the elderly [44]. In the present study, the Harris hip function score and Barthel Index score at 1, 3, 6, and 12 months were lower in the KOA group than in the control group. At the final follow-up, the control group had a significantly higher hip function and ability to perform activities of daily living than the KOA group without needing assistance from a bystander. It suggests that if a patient had an IFs combined with an ipsilateral KOA, their postoperative hip function and ability to perform activities of daily living are significantly reduced. We consider that two factors contribute to this result. First, pain scores account for 44% of the total Harris hip score for hip function, and the most common clinical sign of KOA is pain. At postoperative follow-up, the subjective perception of pain around the knee on the patient’s fractured side may have influenced the outcome of the hip function score, as well as the significant reduction in lower limb mobility due to peri-knee pain, resulting in a lower Barthel Index score. Secondly, patients with KOA will have varying degrees of valgus or deformity, resulting in altered force lines in the lower limb. The hip joint partially compensates for some of the knee joint function to maintain force stability in the lower limb [45, 46]. However, after an IFs in an elderly patient, the hip joint becomes deformed, and the mobility of the limb is reduced due to long-term preoperative compensation, resulting in difficulty in recovering the function of the hip and reduced ability to perform daily activities after surgery.

### Limitations

There were some limitations in this study. The study was retrospective only, and although the essential characteristics of the included cases were similar, bias could not be excluded entirely. This study imposed no interventions on patients with KOA, further refinement is needed in future work. The follow-up period was short, and the sample was tiny and single. Only patients treated with PFNA internal fixation were selected for this study. Later, the sample size and sample type will be increased to investigate further aspects of the relationship between KOA and IFs in the elderly to obtain adequate clinical data.

## Conclusions

In conclusion, in elderly patients with IFs combined with KOA of the fracture side treated with PFNA internal fixation, KOA increases the incidence of postoperative complications of the fracture, prolongs postoperative time out of bed and fracture healing, and reduces postoperative hip function and ability to daily living. Therefore, treating KOA on the fractured side needs to be considered when treating IFs in the elderly.

## Data Availability

The data that support the findings of this study are available from the corresponding author upon reasonable request.

## Abbreviations

KOA: knee osteoarthritis
IFs: Intertrochanteric fractures
PFNA: Proximal femoral nail anti-rotation
K-L: Kellgren-Lawrence
ASA: American Society of Anesthesiologists
SPSS: Statistical Product and Service Solutions
SD: Standard deviation

## References

[1] Tucker A, Donnelly KJ, Rowan C (2018). Is the best plate a nail? A review of 3230 unstable intertrochanteric fractures of the proximal femur. J Orthop Trauma.

[2] Hiragami K, Ishii J (2018). Embedding the lateral end of the lag screw within the lateral wall in the repair of reverse obliquity intertrochanteric femur fracture. J Int Med Res.

[3] Page PR, Lord R, Jawad A (2016). Changing trends in the management of intertrochanteric hip fractures - a single centre experience. Injury.

[4] Socci AR, Casemyr NE, Leslie MP (2017). Implant options for the treatment of intertrochanteric fractures of the hip: rationale, evidence, and recommendations. Bone Joint J.

[5] Yoo JI, Ha YC, Lim JY (2017). Early Rehabilitation in Elderly after Arthroplasty versus internal fixation for unstable intertrochanteric fractures of Femur: systematic review and Meta-analysis. J Korean Med Sci.

[6] Kim EM, Li G, Kim M (2020). Development of a risk score to predict postoperative delirium in patients with hip fracture. Anesth Analg.

[7] Yin C, Zhang J, Er Z (2019). Clinical application of auricular point sticking in perioperative hemostasis for elderly patients with intertrochanteric fractures of the femur. Med (Baltim).

[8] Leung F, Lau TW, Kwan K (2010). Does timing of Surgery matter in fragility hip fractures?. Osteoporos Int.

[9] Zhou S, Liu J, Zhen P (2019). Proximal femoral nail anti-rotation versus cementless bipolar hemiarthroplasty for unstable femoral intertrochanteric fracture in the elderly: a retrospective study. BMC Musculoskelet Disord.

[10] Wang C, Wang Q (2019). Helical blade compression failure occurred during PFNA implantation: a rare case and ingenious solution. Med (Baltim).

[11] Cross M, Smith E, Hoy D (2014). The global burden of hip and knee osteoarthritis: estimates from the global burden of Disease 2010 study. Ann Rheum Dis.

[12] Driban JB, McAlindon TE, Amin M (2018). Risk factors can classify individuals who develop accelerated knee osteoarthritis: data from the osteoarthritis initiative. J Orthop Res.

[13] Heidari B (2011). Knee osteoarthritis prevalence, risk factors, pathogenesis and features: part I. Casp J Intern Med.

[14] Arden NK, Crozier S, Smith H (2006). Knee pain, knee osteoarthritis, and the risk of fracture. Arthritis Rheum.

[15] Altman R, Asch E, Bloch D (1986). Development of criteria for the classification and reporting of osteoarthritis. Classification of osteoarthritis of the knee. Diagnostic and Therapeutic Criteria Committee of the American Rheumatism Association. Arthritis Rheum.

[16] Kang H (2021). Sample size determination and power analysis using the G*Power software. J Educ Eval Health Prof.

[17] LAWRENCE KELLGRENJH (1957). Radiological assessment of osteo-arthrosis. Ann Rheum Dis.

[18] Smith MV, Klein SE, Clohisy JC (2012). Lower extremity-specific measures of disability and outcomes in orthopaedic Surgery. J Bone Joint Surg Am.

[19] MAHONEY F I, BARTHEL D W (1965). FUNCTIONAL EVALUATION: THE BARTHEL INDEX. Md State Med J.

[20] Quinn TJ, McArthur K, Ellis G (2011). Functional assessment in older people. BMJ.

[21] Grill E, Stucki G, Scheuringer M (2006). Validation of International Classification of Functioning, disability, and Health (ICF) core sets for early postacute rehabilitation facilities: comparisons with three other functional measures. Am J Phys Med Rehabil.

[22] Lu Y, Uppal HS (2019). Hip fractures: relevant anatomy, classification, and Biomechanics of fracture and Fixation[J]. Geriatr Orthop Surg Rehabil.

[23] Forni S, Pieralli F, Sergi A (2016). Mortality after hip fracture in the elderly: the role of a multidisciplinary approach and time to Surgery in a retrospective observational study on 23,973 patients. Arch Gerontol Geriatr.

[24] Kang Y, Liu J, Chen H (2019). Enhanced recovery after Surgery (ERAS) in elective intertrochanteric fracture patients result in reduced length of hospital stay (LOS) without compromising functional outcome[J]. J Orthop Surg Res.

[25] Yin M, Yan Y, Fan Z (2020). The efficacy of enhanced recovery after Surgery (ERAS) for elderly patients with intertrochanteric fractures who received Surgery: study protocol for a randomized, blinded, controlled trial. J Orthop Surg Res.

[26] Nherera L, Trueman P, Horner A (2018). Comparison of a twin interlocking derotation and compression screw cephalomedullary nail (InterTAN) with a single screw derotation cephalomedullary nail (proximal femoral nail antirotation): a systematic review and meta-analysis for intertrochanteric fractures. J Orthop Surg Res.

[27] Xu YZ, Geng DC, Mao HQ (2010). A comparison of the proximal femoral nail antirotation device and dynamic hip screw in the treatment of unstable pertrochanteric fracture. J Int Med Res.

[28] Xie Y, Dong Q, Xie Z (2019). Proximal femoral nail anti-rotation (PFNA) and hemi-arthroplasty in the treatment of elderly intertrochanteric fractures. Acta Orthop Belg.

[29] Li H, Zhang W, Yan J (2018). Greater trochanter of the femur (GTF) vs. proximal femoral nail anti-rotation (PFNA) for unstable intertrochanteric femoral fracture. Eur Rev Med Pharmacol Sci.

[30] Chen L, Zheng J, Li G (2020). Pathogenesis and clinical management of obesity-related knee osteoarthritis: impact of mechanical loading. J Orthop Translat.

[31] Mora JC, Przkora R, Cruz-Almeida Y (2018). Knee osteoarthritis: pathophysiology and current treatment modalities. J Pain Res.

[32] Fernandez-Torres J, Martinez-Nava GA, Zamudio-Cuevas Y (2020). Epistasis of polymorphisms related to the articular cartilage extracellular matrix in knee osteoarthritis: analysis-based multifactor dimensionality reduction. Genet Mol Biol.

[33] Ayral X, Pickering EH, Woodworth TG (2005). Synovitis: a potential predictive factor of structural progression of medial tibiofemoral knee osteoarthritis -- results of a 1 year longitudinal arthroscopic study in 422 patients. Osteoarthritis Cartilage.

[34] Kammerlander C, Gosch M, Kammerlander-Knauer U (2011). Long-term functional outcome in geriatric hip fracture patients. Arch Orthop Trauma Surg.

[35] Katz JN, Arant KR, Loeser RF (2021). Diagnosis and treatment of hip and knee osteoarthritis: a review. JAMA.

[36] Glyn-Jones S, Palmer AJ, Agricola R (2015). Osteoarthr Lancet.

[37] Steiner M, Claes L, Ignatius A (2014). Disadvantages of interfragmentary shear on fracture healing–mechanical insights through numerical simulation. J Orthop Res.

[38] Bottlang M, Doornink J, Fitzpatrick DC (2009). Far cortical locking can reduce stiffness of locked plating constructs while retaining construct strength. J Bone Joint Surg Am.

[39] Epari DR, Gurung R, Hofmann-Fliri L (2021). Biphasic plating improves the mechanical performance of locked plating for distal femur fractures. J Biomech.

[40] Shitova AD, Kovaleva ON, Olsufieva AV (2022). Risk modeling of femoral neck fracture based on geometric parameters of the proximal epiphysis. World J Orthop.

[41] Augat P, Merk J, Ignatius A et al. Early, full weightbearing with flexible fixation delays fracture healing. Clin Orthop Relat Res, 1996(328):194–202. 10.1097/00003086-199607000-00031 8653957

[42] Chen X, Yan J, He F (2018). Mechanical stretch induces antioxidant responses and osteogenic differentiation in human mesenchymal stem cells through activation of the AMPK-SIRT1 signaling pathway. Free Radic Biol Med.

[43] Hulth A (1989). Current concepts of fracture healing. Clin Orthop Relat Res.

[44] Chehade MJ, Carbone T, Awwad D (2015). Influence of Fracture Stability on Early Patient Mortality and Reoperation after Pertrochanteric and intertrochanteric hip fractures. J Orthop Trauma.

[45] Fukaya T, Mutsuzaki H, Nakano W (2019). Characteristics of frontal plane lower limb movement during walking in patients with knee osteoarthritis of varying severity. J Orthop Surg (Hong Kong).

[46] Hicks-Little CA, Peindl RD, Hubbard TJ (2011). Lower extremity joint kinematics during stair climbing in knee osteoarthritis. Med Sci Sports Exerc.

